# Perceived Indoor Environment and Occupants’ Comfort in European “Modern” Office Buildings: The OFFICAIR Study

**DOI:** 10.3390/ijerph13050444

**Published:** 2016-04-25

**Authors:** Ioannis A. Sakellaris, Dikaia E. Saraga, Corinne Mandin, Célina Roda, Serena Fossati, Yvonne de Kluizenaar, Paolo Carrer, Sani Dimitroulopoulou, Victor G. Mihucz, Tamás Szigeti, Otto Hänninen, Eduardo de Oliveira Fernandes, John G. Bartzis, Philomena M. Bluyssen

**Affiliations:** 1 Department of Mechanical Engineering, University of West Macedonia, Sialvera & Bakola Str., Kozani 50100, Greece; dsaraga@ipta.demokritos.gr (D.E.S.); sanidimi@gmail.com (S.D.); bartzis@uowm.gr (J.G.B.); 2 Environmental Research Laboratory, INRASTES, National Center for Scientific Research “DEMOKRITOS”, Aghia Paraskevi Attikis, Athens 15310, Greece; 3 CSTB-Centre Scientifique et Technique du Bâtiment, University of Paris-Est, 84 Avenue Jean Jaurès, Marne-La-Vallée 77447, France; Corinne.MANDIN@cstb.fr; 4 Chair Indoor Environment, Faculty of Architecture and the Built Environment, Delft University of Technology, Delft 2628 GA, The Netherlands; celine.roda@gmail.com (C.R.); P.M.Bluyssen@tudelft.nl (P.M.B.); 5 Occupational and Environmental Health Unit, Department of Biomedical and Clinical Sciences “L. Sacco”, University of Milan, Via G.B. Grassi 74, Milan IT-20157, Italy; serena.fossati@unimi.it (S.F.); Paolo.carrer@unimi.it (P.C.); 6 The Netherlands Organisation for Applied Scientific Research (TNO), Delft 49 2600 AA, The Netherlands; yvonne.dekluizenaar@tno.nl; 7 Cooperative Research Centre for Environmental Sciences, Eötvös Loránd University, Budapest H-1117, Hungary; vigami72@yahoo.es (V.G.M.); tamas.szigeti@yahoo.com (T.S.); 8 Department of Health Protection, National Institute for Health and Welfare, POB 95, Kuopio 70701, Finland; otto.hanninen@thl.fi; 9 Institute of Science and Innovation in Mechanical Engineering and Industrial Management, INEGI, Rua Dr. Roberto Frias, Porto 4200-465, Portugal; eof@fe.up.pt

**Keywords:** comfort, indoor air, indoor environmental quality, layout, light, noise, office buildings, open-plan office spaces, perception, thermal comfort

## Abstract

Indoor environmental conditions (thermal, noise, light, and indoor air quality) may affect workers’ comfort, and consequently their health and well-being, as well as their productivity. This study aimed to assess the relations between perceived indoor environment and occupants’ comfort, and to examine the modifying effects of both personal and building characteristics. Within the framework of the European project OFFICAIR, a questionnaire survey was administered to 7441 workers in 167 “modern” office buildings in eight European countries (Finland, France, Greece, Hungary, Italy, The Netherlands, Portugal, and Spain). Occupants assessed indoor environmental quality (IEQ) using both crude IEQ items (satisfaction with thermal comfort, noise, light, and indoor air quality), and detailed items related to indoor environmental parameters (e.g., too hot/cold temperature, humid/dry air, noise inside/outside, natural/artificial light, odor) of their office environment. Ordinal logistic regression analyses were performed to assess the relations between perceived IEQ and occupants’ comfort. The highest association with occupants’ overall comfort was found for “noise”, followed by “air quality”, “light” and “thermal” satisfaction. Analysis of detailed parameters revealed that “noise inside the buildings” was highly associated with occupants’ overall comfort. “Layout of the offices” was the next parameter highly associated with overall comfort. The relations between IEQ and comfort differed by personal characteristics (gender, age, and the Effort Reward Imbalance index), and building characteristics (office type and building’s location). Workplace design should take into account both occupant and the building characteristics in order to provide healthier and more comfortable conditions to their occupants.

## 1. Introduction

Indoor environmental quality (IEQ) can be used to express or describe conditions inside buildings. IEQ encompasses thermal, noise, and lighting conditions, as well as indoor air quality (IAQ) [1]. The way in which occupants perceive indoor environmental conditions may affect their comfort, and consequently their health and well-being, as well as their productivity [2]. While thermal, noise, light, and indoor air quality were associated with occupants’ satisfaction, the relation between indoor environment and occupants’ comfort is not fully understood yet [3,4].

Differences in study design (e.g., building type, building location, construction date, study population, IEQ assessment methods) and statistical analysis methods can lead to mixed findings. IEQ can be assessed through objective physical measurements (e.g., pollutants levels, air temperature, noise levels, and illuminance) and subjective occupant surveys (perception of IEQ by the occupants, satisfaction level). In large scale studies, measurements are often limited due to high-cost of environmental measurements. Subjective measurements (questionnaires) are cheaper, easier to carry out, and can be performed simultaneously across many buildings. Building occupants are considered the “best source of information as regards their needs and comfort requirements” [5]. In US office buildings, analyses of data from a web-based survey administered to 52,980 occupants in 351 office buildings over 10 years by the Center for Built Environment (CBE) showed that acoustics, thermal comfort, and air quality received the lowest satisfaction ratings [5]. Using the Kano’s Model of satisfaction, Kim and de Dear [6] found that “temperature” and “noise” impact negatively on overall satisfaction.

The large number of parameters potentially involved and their interrelationships add complexity to the study of the relations between IEQ and comfort. Besides IEQ, other parameters such as occupants’ control over the indoor environment, amount of privacy, as well as office layout have been linked to occupants’ satisfaction [2,3,4,5,6,7,8,9,10,11]. Building location, type of construction, type and design of the heating, and cooling and ventilation systems applied in buildings were reported as potential factors affecting occupants’ comfort [12]. Changes in office buildings over the last few decades (“tighter” construction structures, increased use of air conditioning and mechanical ventilation, increased use of electronic equipment, replacement of cellular offices by open-plan offices) may alter the relations between IEQ and comfort. Personal factors may also influence the occupants’ overall comfort [5,8,13]. Bluyssen, *et al.* [8], in the frame of the European HOPE project (2002–2005), examined the influence of building, social, and personal factors on the perceived health and comfort in 59 office buildings from 5732 respondents, in eight European countries. As perceived comfort was associated with personal, social, and building factors, perceived comfort would be much more than the average of the perceived indoor air quality, noise, lighting, and thermal comfort. Zalejska-Jonsson and Wilhelmsson’s [12] study revealed that comfort perception presents a variation through important personal characteristics such as age, gender, and occupants’ lifestyle. While a better understanding of occupants’ needs is important for all actors involved in the building process (e.g., designers, engineers, and facility managers), to our knowledge, no study has examined the relations between IEQ and occupants’ comfort in “modern” European office buildings.

Within the framework of the European collaborative OFFICAIR project [14]), this current study aimed to: (i) document how occupants of “modern“ office buildings rate their overall comfort and how they perceive IEQ parameters, (ii) investigate the relations between IEQ parameters perceptions and occupants’ overall comfort, and (iii) examine potential modifying factors of these relations such as personal and building characteristics.

## 2. Materials and Methods

### 2.1. Data Collection

This study, part of the EU FP7 OFFICAIR project, was conducted in “modern” office buildings across Europe. The OFFICAIR study design has been described in detail elsewhere [15]. Briefly, 167 office buildings were investigated between October 2011 and May 2012. The participating countries were Finland, France, Greece, Hungary, Italy, The Netherlands, Portugal, and Spain. Around 20 buildings (10 buildings with at least 30 workers and 10 with at least 70 workers) were selected per country on a voluntary basis and based on the following criteria: (i) new or recently retrofitted buildings (preferably <10 years old); (ii) buildings should have been operating in their current form for a minimum of one year prior to the start of the study (preferably two years); (iii) no major renovations were planned before the autumn of 2012. Additionally, feasibility criteria were considered: access to basic information (e.g., building fabric, services, air conditioning systems (if applicable), cleaning practices, and smoking policies), access to internet for digital questionnaires, and clear point contact. The selected buildings were, as much as possible, diverse in terms of location (urban, rural), ventilation and heating systems, type of offices (cellular *versus* open-plan), types of activities, and facades and floor coverings.

Office workers were asked to fill in a digital questionnaire including questions on perceived IEQ, perceived comfort, health symptoms, as well as individual characteristics, working conditions, and psychological and social aspects. The online questionnaire was available in each local spoken language and it was anonymous. All subjects gave their informed consent for inclusion before they filled in the online questionnaire. The study was conducted in accordance with the Declaration of Helsinki, the protocol was approved by the Ethics Committees of University of Milan (26/2011), University of Porto (34/CEUP/2011), University of West Macedonia (71/18-10-2011), Eötvös Loránd University (OFFICAIR/08-11-2011), Acciona (OFFICAIR/11-10-2011) and the Finnish National Institute for Health and Welfare (396-2011). Furthermore, a checklist was filled in by a trained investigator for each building, gathering information on characteristics of the building (e.g., year of construction, location, *etc.*), mechanical systems (ventilation, heating, cooling), rooms, and activities (e.g., type of work, cleaning schedules).

### 2.2. Self-Reported IEQ

Self-reported IEQ data, collected by questionnaire, were used to investigate occupants’ comfort. Besides an overall perception of each component of IEQ (thermal comfort, noise, light, and air quality), occupants’ perception of detailed aspects of environmental parameters were also collected (air movement, privacy, office layout, office decoration, cleanliness, and the view from windows). The items included in the questionnaire are presented in Table 1. For temperature, air movement, air quality, light, noise, and overall comfort participants were asked: “*How would you describe the typical indoor conditions in your office environment during the past month?*” For amount of privacy, office layout and decoration, cleanliness, and view from the windows, participants were asked: “*How would you describe the following in your office?*” These questions were answered by the occupants using a seven-point scale. The scale was from 1 (dissatisfied) to 7 (satisfied). For those parameters that simultaneously included information about two extreme conditions in contrast (coded −3 and +3) (e.g., ambient temperature (too hot–too cold), temperature variation (too much–not enough), air movement (draughty–still), and air humidity (humid–dry)), responses were converted to new scores on a scale from 1 to 7. In this way, symmetry was assumed (*i.e.,* these two opposite conditions correspond to equal dissatisfaction), which seemed the best possible approach, given the question type. Dissatisfaction levels (values ±3) corresponded to 1, satisfaction level (value 0) corresponded to 7, interim values (±2) and (±1) responded to 3 and 5, respectively.

### 2.3. Covariates: Potential Modifiers

Based on the literature [12], personal (demographic, lifestyle, and working conditions) and building characteristics (building location, year of construction as a surrogate of building construction, type and design of the heating, cooling and ventilation systems) were considered.

Demographic data (age and gender), smoking status (never, former, and current), and working conditions were also collected by the questionnaire. Continuous age was categorized as follows: under 35 years; 36–45; 46–55; and more than 55 years.

The effort/reward imbalance (ERI) index for white collar workers was calculated using the formula provided by Siegrist *et al.* [16]. The ERI model claims that work characterized by both high efforts and low rewards represents a reciprocity deficit between high “costs” and low ”gains”, which could elicit negative emotions in exposed employees. The accompanying feelings may cause sustained strain reactions. So, working hard without receiving adequate appreciation or being treated fairly are examples of a stressful imbalance (ERI > 1) [16,17]. Based on the ERI index two subgroups were defined: stressed (ERI > 1) and not-stressed (ERI ≤ 1) participants. ERI index values less than one show that workers’ efforts are adequately rewarded so they are likely to feel less stressed at their work. On the other hand, values higher than one show that workers’ efforts are not satisfactory rewarded; as a result, they may feel more stressed in their work environment.

Buildings were grouped according to the climate zone of the participating countries: Central/North Europe (Finland, France, Hungary, the Netherlands) and South Europe (Spain, Greece, Italy, Portugal). As the year of construction is an attribute that relates to building characteristics such as type of construction and heating/cooling design, the investigated buildings were classified into three similarly sized groups according to the year of construction: before 1996, between 1996 and 2005, and after 2005. Furthermore, buildings were split according to the presence or not of operable windows. Additionally, buildings were also grouped based on the office type (private, shared, and open-plan offices).

### 2.4. Statistical Analysis

All answers related to satisfaction of IEQ items were averaged, and IEQ items were then ranked.

The relations between all environmental parameters were investigated by Spearman correlation (a strong correlation was defined by a Spearman’s rank correlation coefficient above 0.6). Since “Office layout” was highly correlated with both “Office decoration” (0.66; *p* < 0.001) and “Amount of privacy” (0.64; *p* < 0.001), “Office layout” was included in the regression models.

Proportional odds ordinal logistic regression analysis [18] was used to examine the relations between perceptions of indoor environment parameters and occupants’ comfort. This method has been applied in previous studies [5,12,19]. The dependent variable was the overall comfort satisfaction and the independent (predictor) variables were the satisfaction with each environmental parameter. The dependent variable (overall comfort satisfaction) was expressed in values from an ordinal scale (1 = low satisfaction, 2, 3, 4, 5, 6, and 7 = high satisfaction). The associations are presented as Odds Ratios (ORs) with 95% confidence interval (CI 95%). An OR describes the likelihood of increasing overall comfort satisfaction when one of the predictor variables is increased by one unit while the other variables are kept constant. ORs were used to rank the relations between the indoor environment parameters satisfaction and the overall comfort.

Two models were built to investigate the relations between indoor environment parameters and overall comfort satisfaction. In Model 1, the relations between overall perception of thermal, air quality, light, and noise and occupants’ overall comfort were examined. In Model 2, the relations between detailed indoor environmental parameters and overall comfort were estimated. In particular, perception of the following environmental parameters were examined: “*Too cold or too hot temperature*“, “*Air movement*”, ”*Humid or dry air*”, ”*Stuffy or fresh air*”, ”*Smelly or odorless air*”, ”*Natural light*”, ”*Artificial light*”, ”*Reflection or glare*”, ”*Noise outside the building*”, ”*Noise from building systems*”, ”*Noise within the building (e.g., noise inside the offices like phone calls, colleagues chatting, photocopiers, etc.)*”, ”*Office layout*”, ”*Cleanliness*”, and ”*View from the windows*”. The variable “*Temperature variation*” was not included in the final models due to a lack of significance (*p* > 0.05).

The degree of multicollinearity between independent variables in the regression models was checked by the Variance Inflation Factor (VIF). VIF values were below the value of 10 (values ranged from 1.3 to 1.6 in Model 1, and from 1.1 to 1.5 in Model 2), therefore multicollinearity was not an issue [20]. Goodness of fit of the models was assessed by Pseudo-R squared (*R^2^*) values. Overall, Pseudo-*R^2^* values were over 60% for all selected models, indicating that occupants’ comfort was satisfactorily explained by the models.

Interactions between satisfaction with IEQ and individual/building characteristics were also examined. In case of significant interactions, stratified analyses were conducted to estimate the potential impact of personal and building characteristics on the overall comfort satisfaction.

The statistical significance of each independent variable was tested by the Wald test. The statistics software SPSS version 22 (IBM, New York, NY, USA) was used for the statistical analysis.

## 3. Results

### 3.1. Participation, Characteristics of the Study Population, and Their Work Environment

The number of responses per country ranged between 508 (in Portugal) and 1409 (in Hungary) in a total sample of 7441 questionnaires. The response rate was approximately 40% on average. Distinguishing the countries in two geographical regions, Central/North and South European regions, these percentages were 41% and 59% respectively (Table 2). Respondents varied in relation to their age, education, and type of job. The mean age of the occupants was 40.3 years (standard deviation-SD: 10.1 years), 75% had a graduate or postgraduate degree, and 50% of occupants had a managerial or a professional job.

The respondents worked in buildings located in various areas (urban, suburban, commercial, industrial, rural areas). Overall, 43% of the buildings were constructed after 2005. Most of the buildings had operable windows. Workplaces of the respondents were mainly shared rooms (both shared private or open-plan offices) (78%).

### 3.2. Occupants’ Satisfaction with Comfort and IEQ

In general, the workers were satisfied with their overall comfort (mean: 4.74 where 1 = dissatisfied, 4 = neither satisfied nor dissatisfied, and 7 = satisfied). Overall satisfaction with the different IEQ components was slightly lower, specifically for thermal comfort (mean: 4.48), air quality (mean: 4.12), and noise (mean: 4.26), except for light comfort (mean: 4.89). Concerning the detailed IEQ parameters, office workers were less satisfied with air movement (mean: 3.75), noise within the building (mean: 3.84), and stuffy air (mean: 3.91). The office workers seemed to be satisfied with the other parameters (mean values higher than 4). The highest mean was recorded for the noise outside the building (mean: 5.61) (Table 3).

Overall, 23% of the respondents were dissatisfied with the overall comfort (satisfaction rate lower than 4) in their office. About half of the office workers were dissatisfied with “Privacy” (embedded in the office layout parameter), “Air too dry”, and “Noise inside the building” (percentages were 51%, 46%, and 47%, respectively).

### 3.3. Association between Overall Thermal, Air Quality, Noise Light Satisfaction and Overall Comfort (Model 1)

The OR analysis results of the combined model are summarized in Table 4. The greatest OR value (2.05) was found for “overall noise”. The second main parameter associated with overall comfort was air quality (OR: 1.56). Satisfaction with light and temperature showed almost the same association with overall comfort satisfaction (OR: 1.49 and 1.44, respectively).

### 3.4. Association between the Detailed IEQ Parameters and Overall Comfort (Model 2)

The relations between the overall comfort and the fourteen-detailed indoor environmental parameters are shown in Table 4. The highest OR was found for “Noise inside the building” (OR: 1.43). Layout parameter showed a similar OR (1.41). It should be noted that the layout parameter included information about office decoration and the amount of privacy. Air movement and view from windows were ranked as the weakest variables associated with overall comfort OR (1.04).

### 3.5. Interaction between Individual Characteristics and Satisfaction with IEQ on Overall Comfort

Since significant interactions for gender, age, and ERI index were found, stratified analyses were performed.

#### 3.5.1. Gender

The regression analysis revealed a slightly higher OR for noise and light in men (OR: 2.16 and 1.53) than in women (OR: 1.97 and 1.46). Other parameters showed almost equal ORs between men and women with ORs ranging from 1.44 to 1.60. The same order of IEQ parameters associated with overall comfort in both genders was found (Model 1, Table 5).

Concerning the detailed indoor environmental parameters, there were gender differences in the relations between air movement and the view from the windows and overall comfort: although the associations were significant among men, they were not significant among women. For both genders, noise inside the building and office layout were found to be the most important factors with high ORs. The OR for “Noise from the building systems” was slightly higher in male participants (OR: 1.31) compared to female participants (OR: 1.22). For the other parameters, no gender differences were observed (Table 5).

#### 3.5.2. Age

Whatever the age subgroup, the highest association with overall comfort was found for noise. The highest OR for noise was found in 46–55 years-old subgroup (OR: 2.21) and the lowest OR in <35 years-old subgroup with an OR of 1.96. Concerning air quality, ORs were slightly higher in the youngest and the oldest occupants (OR: 1.60 and 1.63, respectively) as compared to the middle aged occupants (OR: 1.55 and 1.51 for workers aged of 36–45 years and 46–55 years, respectively) (Table 5).

Noise inside the building remained the strongest factor for all age groups except for the group younger than 35 years, where the highest OR was found for office layout (OR: 1.42). Noise within the building and from outside were also important factors for the oldest workers (>55 years-old) (OR: 1.59 and 1.29) compared to the other age subgroups, and office cleanliness showed the highest OR in workers of 46–55 years-old (OR: 1.22) as compared to the other age subgroups. For the age subgroups (≥36 years-old), the parameters of the view from the windows and the air movement were not significantly associated with the overall comfort. In addition to the previous parameters, in age subgroup (>55 years-old), reflection and odor were not significantly associated with the occupants’ overall satisfaction (Table 5).

#### 3.5.3. ERI Index

Workers with an ERI ≤ 1 showed higher ORs for noise (OR: 2.04), light (OR: 1.50), and thermal comfort (OR: 1.46) compared to workers with an ERI > 1. The relation with air quality was higher in workers with an ERI > 1 subgroup (OR: 1.62) (Table 5).

In the subgroup of workers with an ERI > 1, a smaller number of detailed parameters were significantly associated with overall comfort. No significant relation was found for noise from the building systems, reflection, air humidity, odor, windows’ view, and air movement. In both subgroups, the highest ORs were found for noise inside the building and office layout factors, with similar ORs (ranged from 1.40 to 1.47) (Table 5).

### 3.6. Interaction between Building Characteristics and Satisfaction with IEQ on Overall Comfort

Significant interactions for geographical location and office type were found and results of stratified analyses are presented in Table 6.

#### 3.6.1. Geographical Location of Buildings

Highest ORs were found for noise, independently of European region (OR: 2.05 or 2.06). After noise, the order of IEQ items changed according to the study region. Light was the second highest IEQ item (OR: 1.54) in the South European subgroup while air quality was the second highest IEQ item in Central/North European subgroup (Table 6).

Regarding the detailed indoor environmental parameters, slight differences in ORs were observed between the European regions. In Central/North Europe, the highest OR was found for “office layout” (1.42) while “noise inside the building” was the highest association obtained in South European region (OR: 1.47). Furthermore, “air movement” was not significantly associated in offices located in Southern Europe (Table 6).

#### 3.6.2. Office Type

The results showed that noise—which revealed the highest OR of all IEQ parameters, within all subgroups of office type—seemed slightly higher in occupants’ working in private and shared offices (OR: 2.16 and 2.12) than in open-plan offices (OR: 1.95). Slight differences were also observed for light and thermal comfort. Similar ORs between overall comfort and air quality were found in all office subgroups (OR ranged from 1.55 to 1.58) (Table 6).

Concerning detailed IEQ parameters, both noise inside the building and office layout had the highest OR for the subgroup of occupants working in open-plan offices (OR: 1.45). In private offices, occupants were more sensitive to noise from outside (OR: 1.34) as well as to the office cleanliness (OR: 1.27). In the same offices, the fresh air factor had a stronger impact on the overall comfort (OR: 1.30). In shared offices, noise from the building systems showed the highest OR 1.35 (Table 6).

## 4. Discussion

Using data from a cross-European survey, this study examined the relation between perceived indoor environment and occupants’ comfort. Satisfaction with overall noise was the most important IEQ component associated with occupants’ comfort. Specifically, noise within the building (e.g., phone calls, colleagues chatting, photocopiers, *etc.*) and office layout were highly associated with overall comfort. Differences according to personal and building characteristics were identified.

### 4.1. Overall Comfort and Satisfaction with Overall IEQ Components

Office workers in European “modern” office buildings were generally satisfied with IEQ in their work environment, reporting the highest satisfaction score for light comfort and the lowest for air quality.

Concerning the associations between each IEQ component and occupants’ comfort, noise was identified as the most important factor that affects the overall comfort, followed by air quality, light, and thermal comfort. In a previous study conducted in 26 offices (4655 respondents) across five countries over North-Central and South Europe during 1998–1999, thermal comfort was identified as the first factor [3]. Cao *et al.* [10] also reported thermal comfort as the most important parameter in a study conducted in five office buildings (500 respondents) in Beijing and Shanghai. Green buildings, which share design characteristics (e.g., ventilation systems, building envelope, and office layout designs) with modern buildings, are intended to provide optimized indoor environments (minimizing environmental impacts through energy and water conservation measures). Low satisfaction with noise was documented in green buildings. Occupants of 24 office buildings (green and conventional) in Canada completed an online questionnaire related to environmental satisfaction, and although green buildings had a better performance in occupant environmental satisfaction, they had a lower satisfaction with noise and speech privacy [21]. Similarly, higher satisfaction of thermal comfort and lower satisfaction for noise and light aspects was identified in green labeled buildings in the area of Adelaide [22]. Mixed findings may be related to the study design (e.g., sampling site, period, or season) as well as the study population (e.g., building and occupants’ characteristics).

Perceived IEQ is related to building characteristics. In a previous OFFICAIR study [15], the relations between physical building characteristics and comfort at building level were investigated. Comfort was negatively associated with visible mold growth, presence of a documented complaints procedure to report problems related to the indoor environment, and cleaning activities in the evening. Conversely, presence of acoustical insulation and/or sound absorption materials was positively associated with comfort.

### 4.2. Overall Comfort and Detailed IEQ Parameters

Thanks to data collection on satisfaction with detailed IEQ parameters, the identification of the parameters involved in overall comfort was examined more precisely. Concerning noise, “noise within the building” was the most important parameter associated with comfort, followed by “noise from the building systems” and “outside noise”. Concerning air quality, fresh air was the most important parameter, followed by dry air and odor. Natural light was the most important factor for light followed by artificial light and glare. As per thermal comfort, too hot or too cold temperatures were important, while in considering temperature variation, “too much or not enough variation” was less important for the overall comfort.

The highest association with overall comfort was found for noise within the buildings followed by the office “layout, amount of privacy” parameter. Nowadays, office workers often share their workplace in open-plan office rooms. The importance of the noise parameter is in line with Pierrete’s *et al.* [23] who performed a questionnaire study in open-plan offices in which occupants considered that noise in their environment was high. Frontczak *et al.* [5] analyzed data from the CBE and found that amount of space and noise were two important parameters that affect overall comfort. Using the Kano’s satisfaction model, Kim and de Dear [5] showed that these parameters (amount of space and noise) had the biggest effect. Recently, the importance of “amount of space” and “visual privacy through different type of offices” factors was also reported [11]. Aizlewood and Dimitroulopoulou [24] also pointed out privacy is a major aspect in open-plan offices. In our study, amount of privacy, and decoration which was strongly correlated with office layout, were significant attributes that affect overall comfort. Heerwagen [25] also pointed out the importance of decoration inside offices, in which occupants try to improve it with visual materials. View and air movement were seen to be the least important parameters associated with occupants’ comfort.

### 4.3. Overall Comfort and Individual Characteristics

Our results showed that the relations between perception of the IEQ parameters and overall satisfaction vary according to personal characteristics. The relation between comfort and noise was higher in men than in women. Kim *et al.* [26] also documented gender differences in noise level and sound privacy satisfaction. The relations with air quality and noise inside the building were higher in the older occupant subgroup (over 55 years-old). Based on the ERI index, the relations were slightly higher in office workers who felt that their effort is rewarded (ERI ≤ 1) compared to other workers (ERI > 1). Roda *et al.* [27] found that workers with an ERI > 1 reported more building related health symptoms. Therefore, OFFICAIR data highlights the importance to take into account the psycho-social environment in studies examining occupants’ comfort and health.

### 4.4. Overall Comfort and Building Characteristics

In this current study, the relation between comfort and noise, especially noise from the outside, was higher in private offices in which noise, in general, is lower compared to other office types (shared or open-plan offices). On the other hand, in open-plan offices, occupants are affected in a higher degree by the office layout and the amount of privacy and this might be related to the number of occupants per office. This finding is strengthened by Leder *et al.* [28] who studied occupants’ comfort in open-plan and private offices both in conventional and green buildings, where the satisfaction with acoustics and privacy was most strongly affected by workstation size and office type. Pejtersen *et al.* [29] also pointed out that open-plan offices could comprise a risk factor for adverse environment perception and these offices may not be suitable for all job types.

Differences according to European regions were documented. The relation between light and overall comfort was higher in Southern Europe compared to Central/North Europe. This difference may be explained by different climate conditions as well as differences in building construction. Conversely, the relation between air quality and comfort was higher in Northern and Central European offices. Slight differences came out in the Model 2 (*i.e.,* using the detailed indoor environmental parameters).

### 4.5. Strengths and Limitations

This study has several strengths: a relatively large sample size, a survey performed in different geographical areas across Europe, and the use of standardized procedures (questionnaire and checklist). Data on socio-demographics, psycho-social work environment, and perceived environmental quality were collected by a validated questionnaire. IEQ was assessed using both crude IEQ items (satisfaction with overall thermal comfort, noise, light, and indoor air quality), and 14 detailed indoor environmental parameters (e.g., layout, noise within the building, noise from building systems, noise outside the building, air movement). Some limitations should be noted. Caution is needed when interpreting because data on IEQ were self-reported, a potential (recall) bias cannot be excluded, and surveys do not always capture IEQ issues. A combination of objective and subjective measurements would be useful for assessing IEQ in a building. Another limitation is the cross-sectional study design. Therefore, no causality of the identified relations can be confirmed.

### 4.6. Practical Implications

Based on the results of this current study, several practical implications can be noted. Privacy appeared to be an important aspect for office occupants. Speech privacy could be improved by blocking the sound using barrier or partitions between workstations. Adequate amount of space per workstation should be taken into account during the office layout designing as well as the demands for different types of jobs. The type of work or activities to be performed is a key parameter for the design of an office work environment. Allocating spaces for different activities (e.g., making a phone call, having a meeting, or working in silence) requiring some privacy is used more and more, especially in office buildings adopting flex-working resulting in more comfortable workplaces.

Additionally, access to operable windows or local demand control (based on temperature or relative humidity) of the mechanical ventilation system could improve the air quality perception. When applying any form of mechanical ventilation it is important to maintain the components regularly (filters, heating and cooling batteries, heat exchangers, humidifiers). Several recommendations for design and maintenance of such systems and their components are available (e.g., Bluyssen *et al.*) [30].

Although not identified as the most important factor, light conditions—and especially natural light levels—inside the office seem to contribute to the improvement of occupants’ comfort. Appropriate light generally helps in how well occupants can see, and natural light can also affect people’s mood [31]. Consequently, in order to provide healthy and comfortable light, office design becomes an additional challenge, especially in open-plan offices.

## 5. Conclusions

Office workers in European “modern” office buildings were generally satisfied with IEQ in their work environment. The highest satisfaction rating was obtained for light comfort, followed by thermal comfort, noise, and air quality. Satisfaction with overall noise was the strongest IEQ component associated with occupants’ comfort. The relations between perceived indoor environment and occupants’ comfort depend on socio-cultural context, as well as personal and building characteristics. Understanding and evaluating occupants and building characteristics may help in providing healthier and more comfortable buildings.

## Figures and Tables

**Table 1:** List of parameters assessed in the OFFICAIR questionnaire survey.

Parameter	Questionnaire Item
Temperature	Too cold (−3)–Too hot (+3) Varies too much during the day (−3)–Not enough variation (+3) Uncomfortable (1)–Comfortable (7)
Air Movement	Draughty (−3)–Still (+3)
Air Quality	Humid (−3)–Dry (+3) Stuffy (1)–Fresh (7) Smelly (1)–Odorless (7) Unsatisfactory (1)–Satisfactory (7)
Light	Natural Light: Unsatisfactory (1)–Satisfactory (7) Artificial Light: Unsatisfactory (1)–Satisfactory (7) Reflection or glare: Glare (1)–No glare (7) Light Overall: Unsatisfactory (1)–Satisfactory (7)
Noise	Noise from outside the building: Unsatisfactory (1)–Satisfactory (7) Noise from building systems (e.g., heating, plumbing, ventilation, air conditioning): Unsatisfactory (1)–Satisfactory (7) Noise from within the building other than from building systems (e.g., phone calls, colleagues chatting, photocopiers, *etc.*): Unsatisfactory (1)–Satisfactory (7) Noise overall: Unsatisfactory (1)–Satisfactory (7)
Amount of Privacy	Unsatisfactory (1)–Satisfactory (7)
Office’s Layout	Do not like at all (1)–Like very much (7)
Office’s Decoration	Do not like at all (1)–Like very much (7)
Cleanliness	Unsatisfactory (1)–Satisfactory (7)
View from the Windows	Do not like at all (1)–Like very much (7)
Overall Comfort	Unsatisfactory (1)–Satisfactory (7)

**Table 2:** Distribution of buildings and respondents per country and location area in OFFICAIR study.

Country	Spain	Greece	Italy	Portugal	Finland	France	Hungary	The Netherlands	Total Sample
Number of Buildings	20	23	21	19	19	21	24	20	167
Number of Respondents	698	1020	809	508	793	1190	1409	1014	7441
% of Respondents	9%	14%	11%	7%	10%	16%	19%	14%	100%
Location Area	South				Central/North				
% of Respondents	41%				59%				100%

**Table 3:** Description of overall comfort and satisfaction with indoor environmental quality (IEQ) and detailed indoor environmental parameters in OFFICAIR study (item-scale: from 1-Dissatisfied to 7-Satisfied).

Parameters	Number of Respondents	Mean	Standard Error	Confidence Intervals (95%)
Overall Comfort	7366	4.74	0.02	4.70–4.77
*IEQ Components*				
Light Overall	7377	4.89	0.02	4.85–4.92
Thermal Comfort	7137	4.48	0.02	4.08–4.16
Noise Overall	7371	4.26	0.02	4.22–4.30
Air Quality Overall	7178	4.12	0.02	4.08–4.16
*Detailed IEQ Parameters*				
Noise Outside the Building	7378	5.61	0.02	5.57–5.65
Artificial Light	7392	5.03	0.02	4.99–5.07
Too Cold or Too Hot Temperature	7047	5.00	0.03	4.95–5.05
Noise from Building Systems	7368	4.97	0.02	4.92–5.01
Natural Light	7353	4.87	0.02	4.82–4.92
Cleanliness	7387	4.69	0.02	4.65–4.73
Smelly or Odorless Air	7093	4.62	0.02	4.57–4.66
View from the Windows	6929	4.42	0.02	4.38–4.47
Reflection or Glare	7377	4.42	0.02	4.38–4.47
Layout	7394	4.30	0.02	4.26–4.34
Humid or Dry Air	7040	4.16	0.03	4.11–4.22
Stuffy or Fresh Air	7071	3.91	0.02	3.88–3.95
Noise within the Building	7399	3.84	0.02	3.80–4.89
Air Movement	7388	3.75	0.03	3.70–3.80

**Table 4:** Association between overall comfort and satisfaction with indoor environmental quality (IEQ) (Model 1) and detailed indoor environmental parameters (Model 2) in OFFICAIR study.

Variables	Odds Ratios	Confidence Intervals (95%)	
**Model 1**			
Satisfaction with overall Noise	2.05 ***	1.99	2.12
Satisfaction with overall Air Quality	1.56 ***	1.51	1.62
Satisfaction with overall Light	1.49 ***	1.44	1.53
Satisfaction with overall Thermal comfort	1.44 ***	1.40	1.48
**Model 2**			
Noise within the Building	1.43 ***	1.39	1.47
Layout	1.41 ***	1.37	1.46
Noise from Building Systems	1.26 ***	1.23	1.29
Stuffy or Fresh Air	1.26 ***	1.21	1.30
Natural Light	1.19 ***	1.16	1.23
Artificial Light	1.18 ***	1.14	1.22
Too Cold or Too Hot Temperature	1.15 ***	1.12	1.18
Noise Outside the Building	1.15 ***	1.12	1.19
Cleanliness	1.15 ***	1.11	1.18
Reflection or Glare	1.12 ***	1.09	1.15
Humid or Dry Air	1.10 ***	1.08	1.12
Smelly or Odorless Air	1.09 ***	1.06	1.13
View from the Windows	1.04 **	1.01	1.07
Air Movement	1.04 **	1.01	1.06

Results from proportional odds ordinal logistic regression analyses. *** *p* ≤ 0.001; ** *p* ≤ 0.01.

**Table 5:** Relations between overall comfort and satisfaction with indoor environmental quality (IEQ) (Model 1) and detailed indoor environmental parameters (Model 2) according to individuals’characteristics in OFFICAIR study.

OR (95% CI)	Gender		Age				ERI	
	Women	Men	Under 35 years	36–45 years	46–55 years	More than 55 years	Less than or equal to 1	More than 1
**Model 1**								
Noise Overall	1.97 (1.89–2.06) ***	2.16 (2.05–2.26) ***	1.96 (1.85–2.08) ***	2.02 (1.92–2.13) ***	2.21 (2.05–2.38) ***	2.09 (1.90–2.30) ***	2.04 (1.97–2.11) ***	1.90 (1.67–2.16) ***
Air Quality Overall	1.54 (1.48–1.61) ***	1.60 (1.52–1.68) ***	1.60 (1.50–1.69) ***	1.55 (1.47–1.63) ***	1.51 (1.40–1.62) ***	1.63 (1.48–1.79) ***	1.56 (1.51–1.62) ***	1.62 (1.41–1.86) ***
Light Overall	1.46 (1.40–1.52) ***	1.53 (1.46–1.61) ***	1.44 (1.36–1.53) ***	1.53 (1.45–1.60) ***	1.49 (1.39–1.60) ***	1.47 (1.34–1.61) ***	1.50 (1.45– 1.55) ***	1.36 (1.21–1.53) ***
Thermal Comfort	1.44 (1.38–1.50) ***	1.45 (1.38–1.52) ***	1.44 (1.37–1.53) ***	1.45 (1.38–1.52) ***	1.41 (1.32–1.51) ***	1.43 (1.31–1.56) ***	1.46 (1.41– 1.51) ***	1.24 (1.10–1.41) ***
***R^2^***	0.66	0.69	0.62	0.66	0.70	0.72	0.66	0.61
***N***	3544	3330	2047	2625	1411	796	5932	376
**Model 2**								
Noise within the Building	1.41 (1.35–1.47) ***	1.45 (1.39–1.52) ***	1.36 (1.29–1.43) ***	1.42 (1.36–1.49) ***	1.48 (1.39–1.58) ***	1.59 (1.45–1.75) ***	1.42 (1.38–1.47) ***	1.47 (1.31–1.65) ***
Layout	1.42 (1.34–1.4) ***	1.42 (1.35–1.49) ***	1.42 (1.33–1.51) ***	1.40 (1.33–1.48) ***	1.43 (1.32–1.54) ***	1.37 (1.24–1.52) ***	1.40 (1.35–1.45) ***	1.41 (1.24–1.61) ***
Noise from Building Systems	1.22 (1.17–1.27) ***	1.31 (1.26–1.37) ***	1.24 (1.18–1.31) ***	1.28 (1.22–1.34) ***	1.26 (1.18–1.34) ***	1.28 (1.17–1.39) ***	1.28 (1.24–1.32) ***	1.05 (0.95–1.17) (n)
Stuffy or Fresh Air	1.24 (1.18–1.30) ***	1.29 (1.22–1.36) ***	1.26 (1.18–1.34) ***	1.32 (1.25–1.40) ***	1.22 (1.13–1.32) ***	1.16 (1.05–1.29) ***	1.25 (1.21–1.30) ***	1.27 (1.086–1.47) **
Natural Light	1.19 (1.150–1.24) ***	1.19 (1.14–1.24) ***	1.18 (1.12–1.24) ***	1.20 (1.15–1.26) ***	1.20 (1.13–1.29) ***	1.20 (1.09–1.32) ***	1.19 (1.15–1.22) ***	1.26 (1.13–1.40) ***
Artificial Light	1.17 (1.12–1.22) ***	1.20 (1.14–1.26) ***	1.17 (1.11–1.24) ***	1.17 (1.11–1.24) ***	1.18 (1.09–1.27) ***	1.17 (1.06–1.30) **	1.18 (1.14–1.22) ***	1.26 (1.11–1.42) ***
Too Cold or Too Hot Temperature	1.14 (1.11–1.18) ***	1.16 (1.12–1.21) ***	1.14 (1.09–1.19) ***	1.19 (1.14–1.24) ***	1.11 (1.05–1.17) ***	1.13 (1.05–1.22) ***	1.15 (1.12–1.18) ***	1.13 (1.02–1.24) *
Noise Outside the Building	1.16 (1.113–1.21) ***	1.14 (1.09–1.19) ***	1.14 (1.08–1.20) ***	1.15 (1.09–1.20) ***	1.15 (1.07–1.23) ***	1.29 (1.16–1.44) ***	1.15 (1.11–1.18) ***	1.18 (1.06–1.31) **
Cleanliness	1.14 (1.10–1.19) ***	1.16 (1.11–1.22) ***	1.16 ( 1.09–1.23) ***	1.10 (1.04–1.15) ***	1.22 (1.14–1.30) ***	1.14 (1.04–1.26) **	1.14 (1.11–1.18) ***	1.15 (1.02–1.29) *
Reflection or Glare	1.12 (1.08–1.16) ***	1.13 (1.08–1.17) ***	1.09 (1.04–1.15) ***	1.13 (1.09–1.18) ***	1.16 (1.09–1.23) ***	1.07 (0.99–1.17) (n)	1.12 (1.09–1.16) ***	1.07 (0.97–1.19) (n)
Humid or Dry air	1.09 (1.06–1.13) ***	1.11 (1.08–1.15) ***	1.08 (1.04–1.13) ***	1.12 (1.08–1.16) ***	1.07 (1.02–1.12) **	1.14 (1.06–1.22) ***	1.10 (1.08–1.13) ***	1.04 (0.95–1.14) (n)
Smelly or Odorless air	1.09 (1.05–1.14) ***	1.09 (1.05–1.15) ***	1.13 (1.07–1.20) ***	1.08 (1.03–1.14) **	1.08 (1.01–1.15) *	1.09 (0.99–1.19) (n)	1.10 (1.06–1.13) ***	1.07 (0.94–1.21) (n)
View from the Windows	1.03 (0.10–1.08) (n)	1.05 (1.01–1.09) *	1.06 (1.01–1.12) *	1.05 (1.00–1.09) (n)	1.02 (0.96–1.09) (n)	1.02 (0.93–1.11) (n)	1.05 (1.02–1.08) **	0.98 (0.88–1.08) (n)
Air Movement	1.03 (0.10–1.06) (n)	1.05 (1.01–1.08) **	1.05 (1.01–1.09) *	1.03 (0.99–1.07) (n)	1.03 (0.98–1.08) (n)	1.01 (0.94–1.08) (n)	1.03 (1.01–1.06) **	1.03 (0.94–1.14) (n)
***R^2^***	0.60	0.63	0.56	0.61	0.66	0.66	0.60	0.59
***N***	3122	2994	1838	2347	1261	670	5365	331

Results from proportional odds ordinal logistic regression analyses. *** *p* ≤ 0.001; ** *p* ≤ 0.01; * *p* ≤ 0.05; (n) *p* > 0.05.

**Table 6:** Relations between overall comfort and satisfaction with indoor environmental quality (IEQ) (Model 1), and detailed indoor environmental parameters (Model 2) according to building characteristics in OFFICAIR study.

Variables/Subgroups	Country’s Geographical Location		Office Type		
OR (95% CI)	Central/North	South	Private	Shared	Open–Plan
**Model 1**					
Noise Overall	2.05 (1.97–2.14) ***	2.06 (1.95–2.16) ***	2.16 (1.99–2.34) ***	2.12 (1.99–2.25) ***	1.95 (1.86–2.04) ***
Air Quality Overall	1.61 (1.55–1.68) ***	1.49 (1.41–1.57) ***	1.58 (1.47–1.70) ***	1.55 (1.46–1.64) ***	1.55 (1.48–1.62) ***
Light Overall	1.46 (1.41–1.52) ***	1.54 (1.47–1.62) ***	1.49 (1.38–1.60) ***	1.52 (1.43–1.61) ***	1.49 (1.431– 1.56) ***
Thermal Comfort	1.44 (1.38–1.49) ***	1.44 (1.37–1.52) ***	1.44 (1.34–1.54) ***	1.50 (1.41–1.58) ***	1.41 (1.35–1.47) ***
***R^2^***	0.67	0.68	0.68	0.66	0.65	
**N**	4128	2751	1401	2055	3333	
**Model 2**						
Noise within the Building	1.40 (1.35–1.45) ***	1.47 (1.40–1.54) ***	1.44 (1.35–1.54) ***	1.33 (1.26–1.40) ***	1.45 (1.39–1.52) ***	
Layout	1.42 (1.36–1.49) ***	1.40 (1.32–1.47) ***	1.28 (1.18–1.39) ***	1.38 (1.30–1.46) ***	1.45 (1.38–1.53) ***	
Noise from Building Systems	1.27 (1.23–1.32) ***	1.26 (1.20–1.31) ***	1.25 (1.17–1.33) ***	1.35 (1.28–1.42) ***	1.24 (1.19–1.29) ***	
Stuffy or Fresh Air	1.25 (1.19–1.31) ***	1.28 (1.21–1.36) ***	1.30 (1.20–1.42) ***	1.24 (1.16–1.32) ***	1.23 (1.17–1.30) ***	
Natural Light	1.19 (1.15–1.24) ***	1.19 (1.13–1.24) ***	1.17 (1.08–1.26) ***	1.19 (1.13–1.25) ***	1.19 (1.14–1.24) ***	
Artificial Light	1.18 (1.13–1.23) ***	1.18 (1.12–1.25) ***	1.15 (1.07–1.24) ***	1.18 (1.11–1.26) ***	1.20 (1.14–1.26) ***	
Too Cold or Too Hot Temperature	1.16 (1.12–1.19) ***	1.14 (1.10–1.19) ***	1.19 (1.12–1.26) ***	1.13 (1.08–1.18) ***	1.14 (1.11–1.18) ***	
Noise Outside the Building	1.17 (1.13–1.22) ***	1.11 (1.06–1.17) ***	1.34 (1.24–1.44) ***	1.19 (1.13–1.25) ***	1.07 (1.03–1.12) ***	
Cleanliness	1.16 (1.11–1.21) ***	1.13 (1.08–1.18) ***	1.27 (1.17–1.37) ***	1.13 (1.07–1.19) ***	1.13 (1.08–1.18) ***	
Reflection or Glare	1.11 (1.07–1.14) ***	1.14 (1.10–1.19) ***	1.11 (1.04–1.18) ***	1.12 (1.07–1.17) ***	1.13 (1.09–1.18) ***	
Humid or Dry air	1.10 (1.07–1.13) ***	1.09 (1.05–1.13) ***	1.07 (1.02–1.12) **	1.11 (1.07–1.16) ***	1.11 (1.07–1.14) ***	
Smelly or Odorless Air	1.08 (1.03–1.1) ***	1.12 (1.07–1.18) ***	1.12 (1.03–1.20) **	1.08 (1.02–1.14) **	1.11 (1.06–1.16) ***	
View from the Windows	1.03 (0.10–1.07) **	1.06 (1.01–1.11) *	1.09 (1.02–1.16) **	1.03 (0.98–1.08) (n)	1.04 (1.00–1.09) *	
Air Movement	1.06 (1.02–1.08) ***	1.02 (0.98–1.05) (n)	1.03 (0.98–1.09) (n)	1.02 (0.98–1.06) (n)	1.03 (1.00–1.07) *	
***R^2^***	0.61	0.63	0.64	0.57	0.60	
**N**	3686	2430	1258	1863	2930	

Results from proportional odds ordinal logistic regression analyses. *** *p* ≤ 0.001; ** *p* ≤ 0.01; * *p* ≤ 0.05; (n) *p* > 0.05.
